# Bioactive Compounds and Antioxidant Capacity of Valencian Pepper Landraces

**DOI:** 10.3390/molecules26041031

**Published:** 2021-02-15

**Authors:** Eva Martínez-Ispizua, Mary-Rus Martínez-Cuenca, José Ignacio Marsal, María José Díez, Salvador Soler, José Vicente Valcárcel, Ángeles Calatayud

**Affiliations:** 1 Valencian Institute for Agricultural Research (IVIA), CV-315, Km 10.7, 46113 Valencia, Spain; martinez_evaisp@externos.gva.es (E.M.-I.); martinez_mru@gva.es (M.-R.M.-C.); marsal_jos@gva.es (J.I.M.); 2 Valencian Institute for the Conservation and Improvement of Agrobiodiversity (COMAV), Polytechnic University of Valencia, Camino de Vera s/n, 46022 Valencia, Spain; mdiezni@btc.upv.es (M.J.D.); salsoal@btc.upv.es (S.S.); jvalcarc@btc.upv.es (J.V.V.)

**Keywords:** antioxidant activity, ascorbic acid, bioactive compound, carotenoids, landrace, lycopene, phenols, pepper

## Abstract

Sweet pepper is one of the most important economic fruits with nutritional attributes. In this sense, the nutraceutical value of consumed products is a major concern nowadays so the content of some bioactive compounds and antioxidants (phenols, ascorbic acid, lycopene, carotenoids, chlorophylls, and antioxidant activity) was monitored in 18 sweet pepper landraces at two maturity stages (green and red). All the traits except chlorophylls significantly increased in red fruits (between 1.5- and 2.3-fold for phenols, ascorbic acid, and 2-2-diphenyl-1-picrylhydrazyl (DPPH) inhibition activity, 4.8-fold for carotenoid and 27.4-fold for lycopene content), which suggests that ripening is key for obtaining desired fruit quality. Among landraces, P-44 in green fruits is highlighted for its content in carotenoids, chlorophylls, phenols, and ascorbic acid, and P-46 for its antioxidant capacity and lycopene content. Upon maturity, P-48, P-44, and P-41 presented higher levels of phenols and lycopene, and P-39 of phenols, carotenoid, and DPPH. This work reflects a wide variability in the 18 pepper landraces at bioactive compounds concentration and in relation to fruit ripeness. The importance of traditional landraces in terms of organoleptic properties is emphasized as they are the main source of agricultural biodiversity today and could be helpful for breeders to develop new functional pepper varieties.

## 1. Introduction

The key role of diet in preventing illnesses has long been recognized, and people have become more careful with the food they choose to consume by seeking foods with higher nutritional values [1]. This is why many recent research works have focused on the determination and quantification of important bioactive compounds present in plant and food materials.

Traditional varieties or landraces are those that have been differentiated by farmers by means of a historic selection process. Therefore, they represent great genetic heritage as a source of agricultural biodiversity [2], which is a key element for ensuring food quality. In fact, consumer demand for vegetable landraces is increasing worldwide, mainly for their sensory values. Additionally, local varieties are better adapted to specific agroclimatic conditions and are, thus, especially recommended for new kinds of agriculture based on sustainable and low inputs, such as organic production [3,4]. The characterization and use of landraces offer a new chance to improve crop organoleptic quality. This opportunity is applicable to those areas in which genetic erosion resulted in a dramatic loss of biodiversity [5,6] that has derived from agrarian mechanization and prioritization of size uniformity and external fruit aspect when choosing profitable crops for the food industry [7]. However, an opposite trend has recently been set by consumers, who have voiced concern about the organoleptic quality and nutritional value of local products [8,9]. This has led to an increase in the number of research studies that focus on bioactive compound extraction from fruit and vegetables, while studying their impact on the human body [10].

Pepper plants (*Capsicum* spp.) belong to the Solanaceae crop family, formed by vegetables of remarkable agro-economic importance worldwide [11], among other species that make this family one of the most important ones for human diet for their multiple applications [12]. Of the five domesticated *Capsicum* species, the most important from the agronomic point of view is the sweet pepper *Capsicum annuum* L., which has wide phenotypic variability, as well as offering countless cooking applications [13]. Pepper is the second most consumed vegetable worldwide, hence its considerable agronomic and economic importance (1.99 million cultivated hectares (ha) and 38 million tonnes production, 2019) [14]. Furthermore, its nutritional value is important because it is rich in ascorbate (vitamin C), β-carotene (provitamin A), calcium, α-tocopherol (vitamin E), thiamine (B1), riboflavin (B2), niacin (B3), and antioxidants like carotenoids and phenolic acids [15].

The study was carried out in 18 local pepper landraces from the Valencian Community (Spain) that represent a wide phenotype variety. Plant resources were provided by the Valencian germplasm bank (GB) from Valencian Institute for the Conservation and Improvement of Agrobiodiversity (COMAV, Valencia, Spain) and the Valencian Institute for Agrarian Research (IVIA, Valencia, Spain). The aims of this research were: (1) determine the nutritional and nutraceutical characteristics of all the pepper landraces, and (2) to value the most promising materials from a nutritional point of view in order to know the health benefits deriving from their use by considering two maturity stages. This work can contribute to revalue traditional landraces by emphasizing their nutritional values such as added value, and additionally, to enhance certain endangered traditional varieties and promote their use and conservation.

## 2. Results

### 2.1. Nutraceutical Compounds and Antioxidant Capacity

#### 2.1.1. Phenols

**Figure 1:**
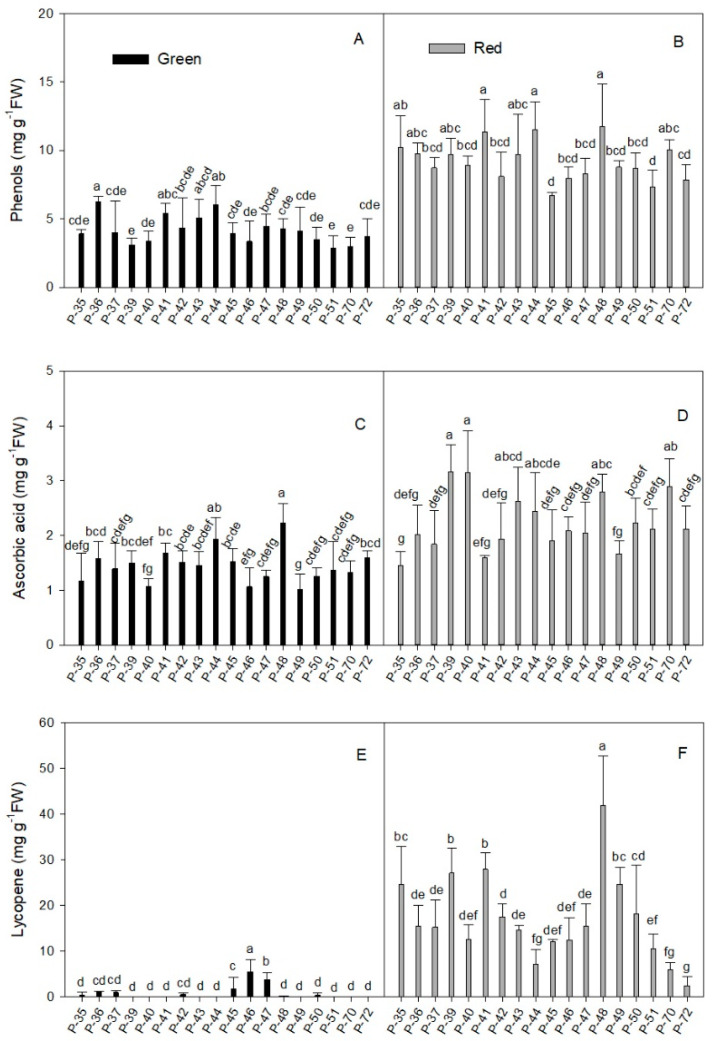
Total phenols (**A**,**B**), total ascorbic acid concentration (**C**,**D**), and lycopene concentration (**E**,**F**) in the green (**A**,**C**,**E**) and red (**B**,**D**,**F**) fruit produced by the 18 pepper landraces. Values are the mean ± SE of four replicates per landrace. Mean is subjected to a one-way ANOVA and different letters indicate significant differences at *p* < 0.05 using the LSD test.

Phenols are phytochemical compounds of interest in pepper fruit for their ability to scavenge free radicals. Table 1 and Figure 1 show the concentration of phenols in green and red fruits of the 18 pepper landraces. Phenol concentration was significantly higher in the red than in the green fruits (mean values of 9.20 and 4.17 mg g^−1^ FW, respectively; Table 1). The mean values of phenols for the different landraces in green fruits ranged between 1.83 and 7.24 mg g^−1^ FW (Table 1). Some landraces (P-36, P-41, P-43, and P-44) stood out for their high phenol concentration (ranging from 5.08 to 6.25 mg g^−1^ FW) (Figure 1A). No differences were found for this trait in the other landraces, whose average phenol concentration was 3.72 ± 0.52 mg g^−1^ FW.

The individual phenol concentration data in the red fruits presented a wider range than in green fruits, while the coefficient of variation in the former was slightly lower than in the latter (21.5% and 34.7%, respectively).

In red fruits, the mean values for the different landraces ranged between 5.66 and 15.87 mg g^−1^ FW (Table 1). The highest phenol values in mature fruits were recorded in four landraces (P-35, P-41, P-44, P-48) whose concentrations ranged from 10.24 to 11.74 mg g^−1^ FW (Figure 1B). The lowest phenol values in red fruits were detected in P-45 and P-51 (6.74 and 7.34 mg g^−1^ FW, respectively).

In addition, although the phenol concentration in all the landraces significantly increased with maturity (2.1-fold on average), some landraces showed more remarkable raises. This occurred with P-35, P-39, P-40, and P-48 (between 2.61- and 3.12-fold) and P-70, which displayed the most marked increase in phenol concentration between red and green fruits throughout the experiment (3.35-fold). In contrast, this rise was only 1.5-fold in landrace P-36 which, despite being the lowest, showed a statistically significant increase between red and green fruits.

#### 2.1.2. Total Ascorbic Acid

Red fruits presented a higher ascorbic acid concentration than the green ones (average of 2.24 and 1.44 mg g^−1^ FW, respectively; Table 1). Both ripening stages presented a similar coefficient of variation (around 29%). Three landraces (P-40, P-46, P-49) in green fruits had the lowest ascorbic acid concentration, close to 1 mg g^−1^ FW (Figure 1C) and it doubled in P-44 and P-48 (1.93 and 2.17 mg g^−1^ FW, respectively). In red fruits (Figure 1D), some landraces (P-39, P-40, P-48, P-70; Figure 1D) stood out for their high vitamin C concentration (between 2.80 and 3.16 mg g^−1^ FW), whereas three landraces (P-35, P-41, P-49) obtained the lowest values (around 1.57 ± 0.11 mg g^−1^ FW). When comparing red and green fruits, the rise in the ascorbic acid concentration in mature fruits was statistically significant (more than 1.7-fold) in several landraces (P-39, P-40, P-43, P-46, P-47, P-50, P-70). The highest increase (three-fold) was recorded for P-40.

#### 2.1.3. Lycopene

Lycopene concentration was higher in red fruits than in their green counterparts (16.98 and 0.62 mg g^−1^ FW, respectively; Table 1). Although mature fruits presented a wider range of individual data, the coefficient of variation was 3.8-fold higher in immature fruits (59.5% and 225.1%, respectively).

The extremely low signal recorded in some extracts of the green fruits suggested that lycopene was almost absent (Figure 1E). This was the case of P-39, P-40, P41, P-43, P-44, P-48, P-49, P-51, P-70, and P-72. In contrast, three landraces (P-45, P-46, and P-47) stood out for having a relatively high lycopene concentration in green fruits (1.75, 3.84, and 2.75 mg g^−1^ FW, respectively). The maturity process improved lycopene content in all the landraces and the average concentration increased by 25.6-fold when comparing red and green fruits (Table 1). In red fruits (Figure 1F), four landraces (P-35, P-39, P-41, P-49) had a lycopene concentration of around 25.0 mg g^−1^ FW, which rose to 41.83 in P-48, which was the highest value (41.83 mg g^−1^ FW). P-44, P-70, and P-72 presented the lowest values (between 2.5 and 7.0 mg g^−1^ FW), which were around three-fold lower than the average for mature fruits.

#### 2.1.4. Carotenoid

**Figure 2:**
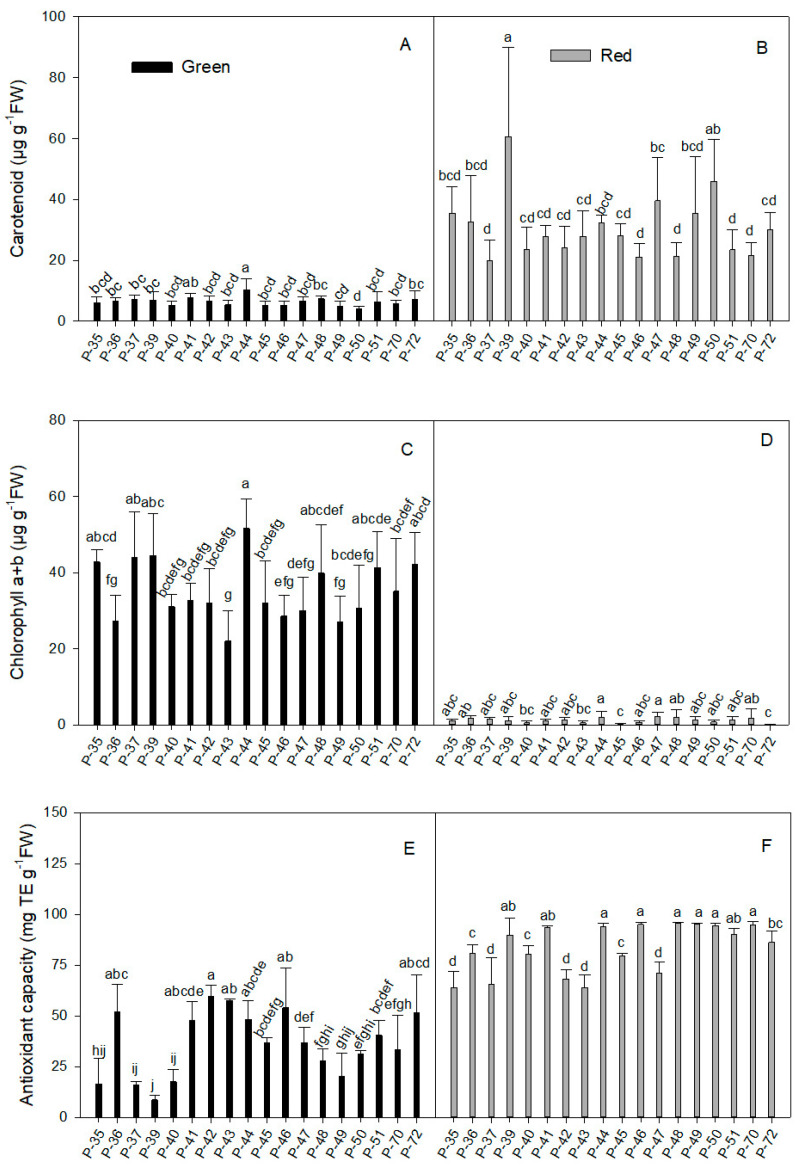
Carotenoid (**A**,**B**), chlorophyll a + b (**C**,**D**), and antioxidant capacity (**E**,**F**) in the green (**A**,**C**,**E**) and red (**B**,**D**,**F**) fruit produced by the 18 pepper landraces. Values are the mean ± SE of four replicates per landrace. Mean is subjected to a one-way ANOVA and different letters indicate significant differences at *p* < 0.05 using the LSD test.

In general terms, carotenoid concentration (Figure 2A) was similar in green fruits from different landraces (average 6.35 µg g^−1^ FW; Table 1). Only P-44 is highlighted for its high value (10.3 µg g^−1^ FW; Figure 2A), while two varieties (P-49 and P-50) had low levels (4.81 and 3.99 µg g^−1^ FW, respectively). Once again, maturity significantly increased carotenoid concentration (4.82-fold; Table 1) and despite the variability in the individual data (45.9% of the coefficient of variation), the average value in most landraces ranged from 21.0 to 35.4 µg g^−1^ FW (Figure 2B). A more marked increase was recorded (8.74-, 7.36- and 11.51-fold) for three varieties (P-39, P-47, and P-50, respectively) when comparing red and green fruits.

#### 2.1.5. Total Chlorophyll Concentration

The total chlorophyll (Chl) concentration was strongly influenced by fruit maturity status (average 35.07 µg g^−1^ FW in green and 1.18 µg g^−1^ FW in red fruits; Table 1). In green fruits (Figure 2C), Chl concentration ranged between 40.23 and 44.53 µg g^−1^ FW in five landraces (P-35, P-37, P-39, P-51, P-72) and rose to 51.65 µg g^−1^ FW in P-44. Three landraces (P-36, P-46, P-49) had very low values (between 27.1 and 28.6 µg g^−1^ FW) and P-43 obtained the lowest value (22.05 µg g^−1^ FW), which was 2.3-fold lower than for P-44.

In red fruits (Figure 2D), two accessions (P-45 and P-72) are highlighted for their very low Chl content (0.15 and 0.08 µg g^−1^ FW, respectively), which was around 7.9-fold lower than the average value of all red fruits included in the experiment (1.18 µg g^−1^ FW; Table 1). However, the highest value for the whole experiment (2.05 µg g^−1^ FW) was for landrace P-44, which was only 1.9-fold higher than the average value.

When comparing the total results between maturity stages, the average Chl concentration (Table 1) in green fruits was 29.7-fold higher than in red ones (35.07 and 1.18 µg g^−1^ FW, respectively). Despite the low data range in mature fruits, the average value was not statistically significant given the high coefficient of variation (93.2%). Among the landraces, the reduction in the total Chl concentration between green and red fruits was between 95% and 97%, and even reached 99% in three varieties (P-40, P-45, and P-72). The least reductions (around 93%) were observed in P-36 and P-47.

#### 2.1.6. Antioxidant Capacity

Antioxidant capacity, as determined by the DPPH assay, was significantly higher in red fruits than in green ones (83.92 and 36.57 mg TE g^−1^ FW, respectively; Table 1). However, immature fruits presented more variability, and the DPPH inhibition range was wider than in mature ones. This was reflected by the coefficient of variation, which was three-fold higher in green than in red fruits (48.9 and 14.77%, respectively). In green fruits (Figure 2E), P-36, P-41, P-42, P-43, P-44, P-46, and P-72 obtained the highest antioxidant capacity values (between 47.56 and 59.69 mg TE g^−1^ FW), while lower values (between 15.95 and 20.4 mg TE g^−1^ FW) were for P-35, P-37, P-40, and P-49, which dropped to 8.6 mg TE g^−1^ FW in P-39, which was the lowest value observed in the experiment. In red fruits (Figure 2F), the antioxidant capacity of several landraces ranged from 89.9 to 95.58 mg TE g^−1^ FW (P-39, P-41, P-44, P-46, P-48, P-49, P-50, P-51, P-70). Only five landraces (P-35, P-37, P-42, P-43, P-47) presented low DPPH inhibition (around 66.4 ± 3.5 mg TE g^−1^ FW). When comparing green and red fruits, the lowest but statistically significant increase was detected in P-36 (1.5-fold). DPPH inhibition capacity did not significantly change in landraces P-42 and P-43, but they both had the least increased values (1.14- and 1.11-fold, respectively).

As previously mentioned, as all the landraces turned red upon maturity, the increased lycopene inside landraces was evidenced even in those in which this pigment was not recorded in green fruits. However, several landraces are worthy of a special mention, such as P-48 because it had the highest lycopene concentration in red fruits (41.83 mg g^−1^ FW), or P-39, P-41, and P-49 with around 27.1 mg g^−1^ FW, despite it being absent in the green ones. It is also worth noting P-46 because it exhibited a higher concentration in green fruits but recorded one of the lowest lycopene increments upon maturity (only 2.2-fold).

### 2.2. Nutraceutical Compounds and Antioxidant Capacity Correlations in Green and Red Fruits

**Figure 3:**
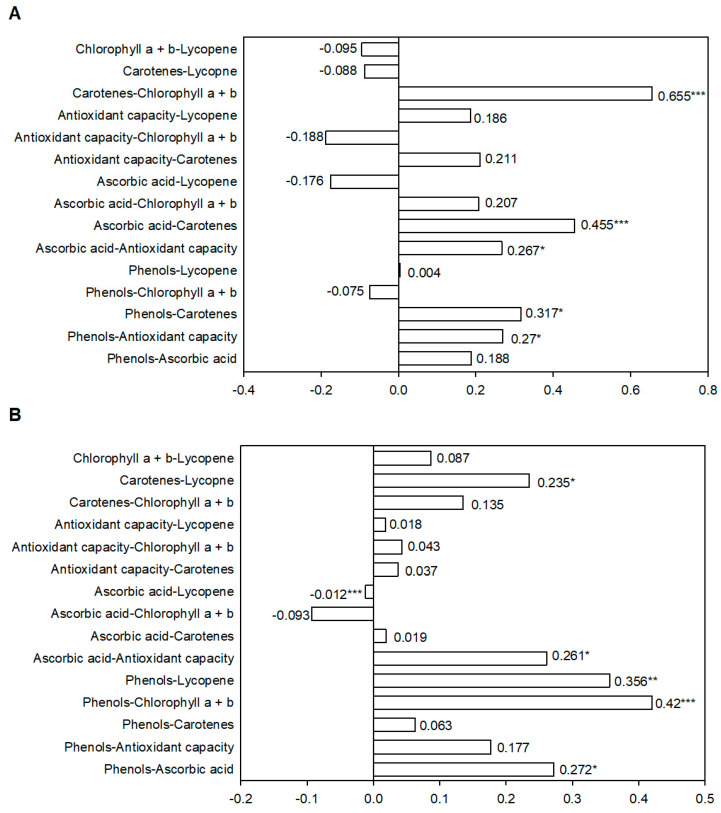
Linear correlation coefficient (r) and its significance for fruit traits (**A**) in green and (**B**) red fruits in the collection of the 18 pepper landraces cultivated in Spain. ***, ** and * indicate significance at *p* < 0.001, *p* < 0.01, *p* < 0.05, respectively.

To understand the contribution of different phytochemicals and antioxidant capacity in fruits at both maturity stages, several correlation analyses were carried out with the different combinations of the six traits (Figure 3).

In green fruits, pairwise coefficients showed a positive correlation and statistical significance for five trait combinations (Figure 3A). The highest correlations were related to carotenoid content. The combination carotenoid vs. total Chls obtained the highest r-value (r = 0.655), while the correlation coefficients for carotenoid vs. ascorbic acid and vs. phenols were 0.455 and 0.317, respectively. Despite the low values (around r = 0.27), antioxidant capacity also positively related to phenols (r = 0.27) and ascorbic acid (r = 0.267). Lycopene did not correlate significantly with any trait in green fruits.

In red fruits (Figure 3B), five positive and statistically significant correlations were found for the 15 studied combinations. The two highest pairwise correlations were for phenols vs. chlorophylls and for phenols vs. lycopene (r = 0.420 and r = 0.356, respectively). Lycopene also correlated with carotenoid concentration in mature fruits (r = 0.235). Antioxidant activity and phenol concentration both correlated with ascorbic acid concentration (r = 0.261 and r = 0.272, respectively). In this case, a negative and statistically significant correlation was found between ascorbic acid and lycopene concentration, despite its low r-value (r = −0.012).

### 2.3. PCA Analysis

The PCA analysis and eigenvalues above one reflected a different pattern in the correlation of the traits in both the green and red fruit (two PCs and three PCs, respectively).

In green fruits, the first and second PCs accounted for 43.3% and 28.82% of the total variation for the studied six traits, respectively (Table 2). The first PC correlated positively with all the traits, except for lycopene content. Two traits (ascorbic acid and carotenoid content) showed the highest values for the correlation of the first PC (0.54 and 0.575, respectively). Phenols and chlorophylls presented moderate correlations (around 0.38), while antioxidant capacity had a low value (<0.15). A low value (−0.25) was obtained for the negative correlation of the first PC with lycopene content. When analyzing the second PC, the highest positive correlation was recorded for antioxidant activity (0.64). A moderate positive correlation was found with phenol and lycopene concentrations (0.46 and 0.36, respectively). In this case, two traits presented a negative correlation with the second PC, a moderate value was obtained for chlorophyll content (−0.50) and carotenoid content came very close to zero (−0.027).

**Figure 4:**
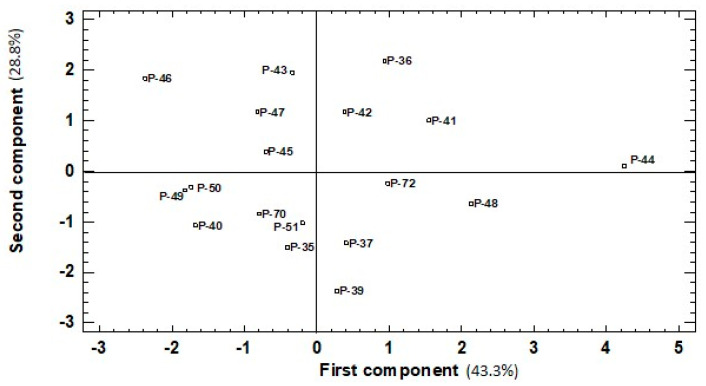
Similarities among green fruits belonging to the 18 pepper accessions evaluated based on six traits (total phenolics, ascorbic acid, carotenoid, lycopene, and chlorophyll content, DPPH scavenging activity) represented the two first components (first component, x-axis; second component, y-axis) of the principal components analysis (43.3% and 28.8% of the total variation, respectively).

In red fruits, the first, second, and third PCs accounted for 33.6%, 20.3%, and 17.1%, respectively. The first PC correlated positively with all the traits included in the analysis. The highest values (>0.4) for the correlation of the first PC were for phenols, chlorophyll, and lycopene concentration, while moderate correlations (between 0.2 and 0.3) were found with the other traits. The second PC correlated significantly and negatively with ascorbic acid and DPPH (−0.66 and −0.50, respectively), and moderately with carotenoid content (−0.32). This second PC showed a moderate positive correlation (0.35 and 0.27) with the other pigments (chlorophylls and lycopene, respectively) and had a very low value with phenols (0.14). The third component showed a positive correlation with two traits: a strong one with carotenoid content (0.81) and a moderate one with lycopene (0.38). The negative correlation with the other four traits ranged from −0.27 to −0.16. For both ripening stages, the projection on the PCA plot showed that landraces were widely spread over the area. In green fruits, four to six landraces were found plotted in each quadrant (Figure 4). The highest value for the first PC was recorded for landrace P-44 (Figure 4) and correlated with its top levels for four traits: phenols, ascorbic acid, carotenoids, and chlorophylls, (Figure 1A,C and Figure 2A,C). P-46, with the lowest value for the first PC, came in the last-but-one place for carotenoids, chlorophylls, and ascorbic acid traits, and the fourth-lowest place was for phenols, albeit not statistically significant compared to the lowest values. Nevertheless, P-46 had one of the highest values for the second PC given its high DPPH and lycopene levels (Figure 1E and Figure 2E). Two more landraces (P-36 and P-43) presented high values for the second PC because of their high phenol concentration and antioxidant capacity (Figure 1A,E), but low chlorophyll and lycopene contents (Figure 2C,E). The landraces with the lowest value for the second PC (P-35, P-37, and P-39) displayed the least DPPH capacity (Figure 2E) and their phenols concentration (Figure 1A) did not statistically differ from the lowest ones. However, their high Chl concentration (rank 4, 2, and 3, respectively) did not significantly differ from the highest value recorded for P-44 (Figure 2C).

**Figure 5:**
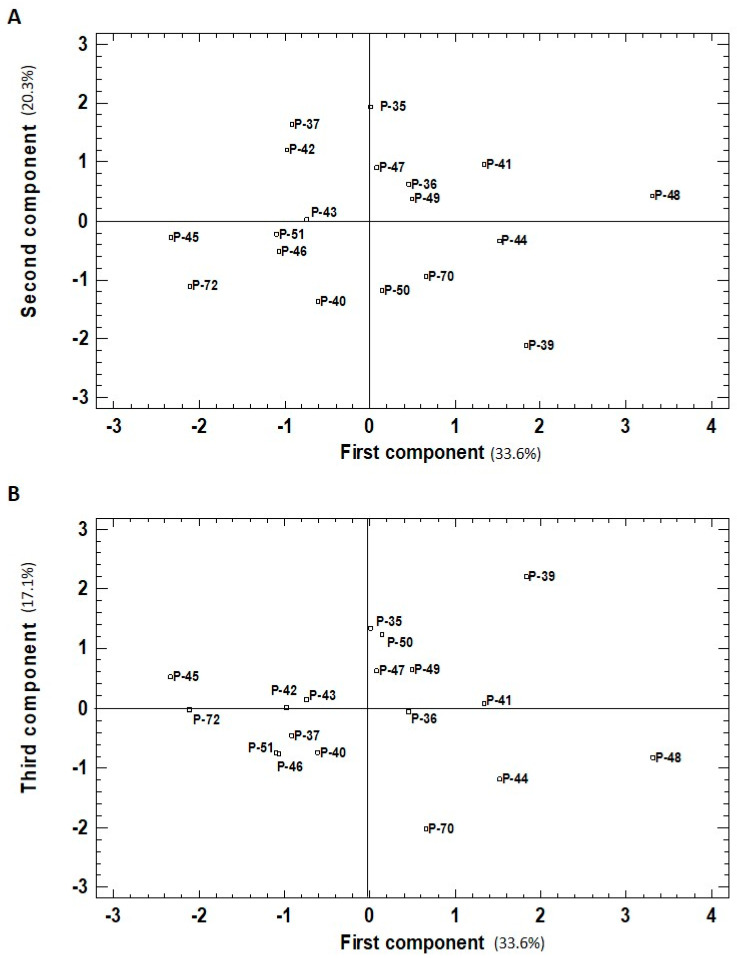
Similarities among red fruits belonging to the 18 pepper accessions evaluated based on six traits (total phenolics, ascorbic acid, carotenoid, lycopene and chlorophyll content, DPPH scavenging activity) represented in (**A**) the two first components (first component, x-axis; second component, y-axis) of the principal components analysis (33.6% and 20.3% of total variation, respectively); and (**B**) the first and third components (first component, x-axis; third component, y-axis) of the principal components analysis (33.6% and 17.1% of the total variation, respectively).

The PCA results in red fruits revealed that the first PC separated the landraces with the highest phenols, chlorophyll, and lycopene contents (Figure 5). P-48 obtained the first highest value for this component as it ranked in first place for phenols and lycopene concentrations, and third for chlorophylls. Three other landraces (P-44, P-41, and P-39) with a high value for the first PC also displayed good levels of phenols (rank 2, 3, and 7, respectively), but were not statistically different from the P-48 level (Figure 1B). P-44 had the second-highest value for chlorophylls, and P-41 and P-39 had the second and third highest lycopene contents. The two accessions with the lowest values for the first PC (P-45 and P-72) also had the lowest values for phenols (rank 18 and 16, respectively; Figure 1B), chlorophylls (rank 17 and 18, respectively, Figure 2D), and lycopene (rank 14 and 18, respectively, Figure 1F).

As the second PC strongly and negatively correlated with ascorbic acid and DPPH activity, the landraces with the highest values for this component presented very low levels for both these traits (Figure 5A). This was the case of P-35, with the lowest levels of both determinations in red fruits for the whole experiment (Figure 1D,F). Three other landraces (P-37, P-42, and P-47) were among the lowest values for phenols (rank 14, 15, and 11, respectively; Figure 1B) and DPPH capacity (rank 15, 14, and 13, respectively; Figure 2F). In contrast, the lowest values for the second PC, recorded for P-39 and P-40, related with their high phenol content in red fruits (rank 1 and 2, respectively, Figure 1B). P-39 also has good levels of carotenoid (rank 1, Figure 2B) and DPPH (rank 8, but with the same significance as the highest values; Figure 2F). The position of P-50 for this second PC was related to its high carotenoid and DPPH capacity, with rank 2 (Figure 2B) and 4 (Figure 2F), respectively.

The third PC of the analysis in red fruits correlated with a high level of carotenoid concentration and moderate lycopene concentration (Figure 5B). The landrace with the highest value for this component (P-39) ranked 1st and 3rd, respectively, for these traits (Figure 1F and Figure 2B). Two other landraces with good levels of the third PC were P-35 and P-50, with rank 4 and rank 2 for carotenoids (Figure 2B), and rank 5 and 6 for lycopene, both respectively (Figure 1F). Instead, P-70 was that with the lowest third PC value and low contents of both traits (rank 15 and 17, respectively).

### 2.4. Differences between Accession Groups

According to the PCA analysis, the 18 accessions were located on the plot and grouped according to their first/second PCs distribution and positive/negative values (+/−). Groups A (positive for both components, +/+), B (+/−), C (−/−), and D (−/+) were formed. The statistical analysis performed among the four groups detected significant differences in all the studied traits (Table 3).

In the green peppers, the first vs. the second plot (Figure 4) showed that group A stood out for the highest phenol values, which agreed with the fact that it included three of the four accessions with the highest values for this parameter in green fruits (P-36, P-41, P-44; Figure 1A). Group A, together with group B, also showed the highest ascorbic acid values (one of the two landraces with the highest values; P-44; Figure 1C), DPPH (six of seven; P-36, P-41, P-42, P-43, P-44, P-46; Figure 2E). Two landraces in these groups (P-41 and P-44) had the highest carotenoid content compared to the other studied varieties (Figure 2A). In general, group C grouped those landraces whose phenolic, ascorbic acid, and carotenoid contents were very low, and also included four of the seven landraces with the lowest antioxidant capacity. However, landraces P-35 and P-51 stood out for their high chlorophyll concentration in green fruits. Group D included two of the three landraces (P-46 and P-47) with the highest lycopene concentration in green fruits (Figure 1E).

In the red peppers, the first vs. second plot (Figure 5A) showed that groups A and B included five of the eight landraces with the highest phenols content (P-36, P-39, P-41, P-44, P-48; Figure 1B) and 7 of 14 with chlorophyll concentration (all except P-72; Figure 2D). The landraces from group B had the highest ascorbic acid levels (three of six; P-39, P-44, P-70; Figure 1D). The four landraces in this group were among the seven with the highest antioxidant capacity. The three landraces from group D are among the four with the lowest DPPH capacity in the red fruit. From the third vs. the first plot (Figure 5B), group A included five of the seven landraces with the highest carotenoid concentration in red fruits (P-35, P-39, P-47, P-49, P-50; Figure 2B). Groups C and D included those landraces with the lowest concentrations in chlorophyll, six landraces with the lowest phenol values (6 of 10; P-37, P-40, P-45, P-46, P-51; P-72; Figure 1B), and lycopene (five of six; P-40, P-45, P-46, P-51, P-72; Figure 1F).

## 3. Discussion

The chemical composition, particularly that of nutraceutical compounds, of a vegetable landrace can confer the product added value, which falls in line with increased consumer concern about the nutritional and nutraceutical values of products and their positive relation to human health [16,17,18]. Thus, the high antioxidant capacity of pepper fruit, together with its marked richness in ascorbic acid, carotene, phenols, xanthophylls, and flavonoids, make it functional food [19,20,21].

Of all bioactive constituents, ascorbic acid, lycopene, and phenols are of much interest for pepper quality and depend on both landrace and ripening stages. In our study, red fruits presented almost two-fold more phenolic compounds than green ones, as previously reported in other pepper landraces [19], grafted pepper [1,22,23], and other crops such as tomato [24]. The mean values ranged between 6.74 and 11.74 mg g^−1^ FW in red fruits (Figure 2B) and decreased between 2.88 and 6.25 mg g^−1^ FW in green ones (Figure 2A), which are higher than those reported by several authors [4,20,25,26] for red peppers, but similar to those found by Chavez-Mendoza et al. [22] and Sun et al. [27]. Shaha et al. [28] reported that a gradual increase in phenolic concentration was observed from green to red ripening. Kevers et al. [29] also reported high levels of total phenols in red, yellow, and green peppers (296, 284, and 215 mg 100 g^−1^ FW, respectively), which are even higher than those found in spinach, broccoli, cucumbers, and carrots. Conversely, Rodriguez-Burruezo et al. [30] and Vera-Guzmán et al. [31] characterized several landraces from Spain, Mexico, and the USA for bioactive compounds, and obtained a wide range of variation. Navarro et al. [32] did not find any differences in phenols concentration between green and red fruit from cv. Orlando, a “California”-type pepper obtained from a commercial nursery. Blanco-Ríos et al. [33] discovered that the green bell pepper had the highest total phenol content, but no significant differences between red, yellow, and orange were observed. According to Marín et al. [34], the immature fruit contained the highest phenolic concentrations, while ripe fruit contained the lowest. The intrinsic landrace effect is worth highlighting as we observed that not always do the landraces with the highest total phenols content upon maturity correspond to those with the highest relative increase between green and red fruit. In addition, low phenol levels do not necessarily mean poor antioxidant capacity because this also depends on the phenolic compounds profile, as suggested by some authors [35,36]. In our study, a positive correlation was obtained between both traits in green fruits.

Otherwise, the free radical scavenging abilities of peppers determined by the—DPPH method were 2.29-fold lower for green than red fruits. However, green fruits presented more variability and a mean DPPH inhibition for the different landraces, which ranged from 8.58 to 59.69 mg TE g^−1^ FW (Figure 2E). DPPH inhibition in red fruits ranged only from 63.92 to 95.58 mg TE g^−1^ FW (Figure 2F). These results evidenced that regardless of landrace, red fruits had the highest antioxidant capacity compared to green ones, which coincides with data also reported by other authors in sweet bell pepper cultivars [26,33]. When comparing antioxidant capacity between fruits inside the same landrace, we observed that antioxidant capacity was generally positively influenced by ripening. The most marked increase (10.5-fold) was found in P-39, while no significant increment in antioxidant capacity took place in P-42 and P-43. The influence of maturity processes on DPPH activity has also been reported by Sun et al. [26] who attributed the difference in antioxidant activity between green and red peppers to their different carotenoid, phenolic, and flavonoid contents.

However, the correlation analysis in the whole of fruits only revealed a significant correlation between antioxidant capacity and two parameters (phenolic and vitamin C contents) in green fruits (Figure 3A). Both correlation coefficients were very low (r = 0.270 and r = 0.267, respectively), which was likely due to the wide range of the features and variability in the landraces herein used. There is no defined trend to correlate antioxidant capacity and phenolic content as different authors’ results are extremely variable. Although positive correlations have been determined in date, apple, and pear cultivars [37], non-significant interaction has been reported in cereal crops [38]. These differences could be associated with the diverse responses of phenolic compounds in the Folin–Ciocalteau method [39]. Moreover, not all of the many possible phenolic compounds are active radical scavengers or exert an identical matrix effect [40]. The correlation results were more variable in the red fruits (Figure 3B), which suggests that pepper fruit’s antioxidant activity may also be attributed to other soluble compounds besides polyphenols. This was the case of, for instance, lipophilic compounds with antioxidant properties (e.g., most carotenoids) that assays usually ignore [35].

We also determined lycopene and total carotenoid content. Although the concentration of lycopene showed a marked variability between plant materials for both ripening stages, it was especially notable in immature fruits as reflected by the coefficient of variation (Table 1). While the low signal recorded in some green extracts suggested a lack of lycopene, three landraces stood out for their relatively high lycopene concentration in green fruits. This was the case of P-45, P-46, and P-47 (Figure 3E) with 1.80, 5.50, and 3.84 mg g^−1^ FW, respectively. All the landraces had high lycopene levels in red fruits (around 27.38-fold higher than green ones; Table 1), even those with a low or practically no signal in immature fruit (Figure 3E). Although this was an expected result, several varieties are worth mentioning. One is P-48, which had the highest lycopene concentration in red fruits (41.83 mg g^−1^ FW). Landraces P-35, P-41, P-39, and P-49 (26.05 ± 1.73 mg g^−1^ FW) had the second-highest levels of this compound, although it was practically absent in green fruits. In contrast, P-46 showed a minor lycopene increment upon maturity despite its highest level in green fruits. All these results support the notion of the wide variability in the behavior of landraces toward the maturity process, as previously mentioned, and agrees with other studies conducted with many commercial pepper cultivars, such as bell and California-type pepper plants, and with other species such as tomato [1,22,32,41]. In addition, the possible origins of lycopene must also be considered as the bibliography does not clarify a single one, but suggests that ripening-dependent lycopene accumulation may derive from either β-carotene synthesis inhibition or an alternative ripening-specific pathway like the 1-deoxy-D-xylulose-5-phosphate pathway [42,43]. In the tomato, it is not known whether the progressive transition in pulp color from red to orange–yellow, which signifies over-ripening, derives from the conversion of accumulated lycopene into β-carotene, or from senescence-related lycopene degradation [43,44]. Nevertheless, our results about lycopene are most interesting and relevant for several reasons: (1) the most efficient quencher of singlet oxygen and free radicals among carotenoids [45]; (2) unlike β-carotene, lycopene is used entirely as an antioxidant because it is not transformed into vitamin A [46]; (3) it is well-known that lycopene is not sensitive to heat treatment, such as ascorbic acid and, thus, remains unaltered even after cooking fruit [41].

Regarding the total carotenoids concentration, although we observed differences in landraces, this compound apparently depends less on plant material, which is the exact opposite of lycopene. In general terms, most landraces presented a similar carotene concentration in both green (around 6.35 µg g^−1^ FW) and red (about 30.58 µg g^−1^ FW) fruit. Only P-44 in green fruits and P-39, P-47, and P-50 in red fruits are highlighted for their high carotene content in relation to the corresponding average value for each maturity stage (between 2- and 3.5-fold higher, respectively). Differences between plant materials have been reported in other studies carried out with chili and sweet pepper landraces [31,47] and grafted pepper plants [22,23,48]. We observed a marked dependence for carotenoids on fruit ripeness, which was stronger in fully ripe than in immature fruits. Carotene concentration was around 4.2-fold higher in red than in green fruits in most landraces, but this behavior rose between 7.36- and 11.51-fold in three of the 18 landraces (P-39, P-49, P-50). This finding in mature fruits has been reported by several authors in mature fruit, specifically commercialized paprika, and sweet and hot chili peppers [32,33,49,50] and is related to not only the increment in the number of total carotenoids, but also to the change in the pigment profile [4,34]. During pepper ripening, chloroplast pigments (chlorophylls and carotenoids like lutein and neoxanthin) disappear, while carotenoid chromoplast pigments (β-carotene and xanthophylls like capsanthin) are synthesized [34,51,52,53]. Our mean values of total chlorophylls in all the landraces support these works because the green fruits obtained a high total chlorophyll content in them all (between 27.10 µg g^−1^ FW and 51.65 µg g^−1^ FW), which lowered in the red fruits by more than 95% as a consequence of the ripening process, and concomitantly with an increment in carotenoids. Conversely, Sun et al. [40] have reported a similar carotene concentration between green and red bell peppers. However, this result is not comparable to ours because the fruit belongs to different varieties.

Interestingly, some reports have observed substantial variations for many carotenoids in colored fruit from traditional landraces, which suggests reservoirs of useful traits, including those that might be able to contribute to improved human nutrition and new breeding opportunities [54]. However, Tripodi et al. [55] demonstrated a wide range of bioactive compounds in pepper, including carotenoids, were highly dependent on the environmental component.

In short, we observed that the increase in both total carotenoid and lycopene content did not necessarily display the same tendency. This was supported by the lack of a significant correlation between carotenoids and lycopene in green fruits, and by the statistically significant, but low, r coefficient in red ones (r = 0.235, Figure 3B). Similar results have been reported by Chávez-Mendoza et al. [22], who found an increase in both fruit antioxidant capacity and β-carotene content, but not in lycopene content, with two pepper cultivars when they were grafted onto rootstock “Terrano”.

Finally, the variability of vitamin C between landraces suggests genotype dependence for this trait in peppers. The average ascorbic acid concentration in the different landraces ranged from 1.07 to 2.23 mg g^−1^ FW in green fruits (Figure 2C), and from 1.45 to 3.16 mg g^−1^ FW in red ones (Figure 2D). These values are similar to others reported in studies conducted in commercial sweet pepper and chili cultivars [56,57] and other traditional pepper ecotypes [30]. Higher values have also been recorded by Osuna-García et al. [58] and Palma et al. [59] in their studies carried out in sweet pepper and chili varieties, and also by Antonius et al. [26], Orobiyi et al. [60], and Ribes-Moya et al. [4] in traditional landraces, especially red fruit.

When comparing green and red fruits, some landraces are highlighted because their ascorbic acid concentration depended on the ripening stages, with statistically higher values in the mature ones than in the immature ones. This is the case of landraces P-39, P-43, P-46, P-47, P-50, and P-70, whose mean vitamin C content was between 1.63- and 2.18-fold higher in red than in green fruits, and P-40 with the highest increment (2.95-fold) throughout the experiment. The landraces with the highest values in immature fruits did not obtain the highest relative increments upon ripening. For instance, P-40 and P-70 had the lowest values throughout the experiment in green fruits (1.07 and 1.33 mg g^−1^ FW, respectively), but the highest ones (3.15 and 2.89 mg g^−1^ FW, respectively) upon maturity. We also found that some landraces, such as P-35 and P-49, had low ascorbic acid levels for both maturity stages and displayed a non-significant increment for mature fruits. All these findings indicate a marked dependence of this compound on landrace, which agrees with other studies carried out on traditional pepper varieties [30,61,62]. The relation between ascorbic acid and fruit ripening, particularly the increment of this nutraceutical compound in fresh peppers as fruit advanced, has been previously described in commercial cultivars [32,33,34,36,58,63,64]. Palma et al. [59] did not find any differences in ascorbate concentration upon maturity in Melchor and Piquillo varieties, but they did in Padrón and Alegría. These authors pointed out a likely stabilizing role of ascorbate to assure capsaicinoids levels when oxidized by peroxidases during maturity.

Moreover, the vitamin C levels found in these landraces in both red and green fruits support the notion that pepper is one of the crops with the highest levels of this compound. According to the FAO (Food and Agricultural Organization) and the WHO (World of Health Organization) recommendations, fruits with more than 1.13 mg g^−1^ FW are rich in vitamin C (as in all red peppers fruits and most green ones; Figure 2C,D) and can be considered potential vitamin C sources, as previously reported by Orobiyi et al. [60]. In fact, pepper had similar levels to those of other vegetables that are well-known for their vitamin C content, such as kale or broccoli [65], or even more than double that found in fruits like citrus, grapevine, kiwi fruit, or strawberry [66]. As an average intake of 25 mg of ascorbic acid is enough to meet the daily intake of this vitamin in humans [67], 50 g of fresh fruit intake from most local analyzed landraces would provide such requirements, even when unripe. Thus, the nutraceutical value of pepper might not be questioned.

With the correlation analysis (Figure 4), we found a varied range of correlations among the studied nutraceutical compounds. Generally, ascorbic acid and carotene contents are likely the two nutraceutical compounds with the most marked relations with the other compounds. In green fruits, carotenoids positively correlated with phenols, chlorophylls, and ascorbic acid. This last compound was also positively related to antioxidant capacity in immature fruits. The acceptable concentration of carotenoids found even in green fruits suggests an active synthesis-degradation route of β-carotene toward other successor compounds, such as capsanthin or capsorubin, which are exclusive of pepper and have antioxidant properties, or vitamin A, an essential nutrient not produced by the human body, but one essential for growth and development, epithelial tissue maintenance, reproduction, and proper visual system functioning in the regeneration of photoreceptors [68].

DPPH positively correlated with phenols in green fruits, but not in red fruits, while antioxidant capacity and phenols seem to be related more to ascorbic acid concentration in mature fruits, which indicates a strong dependence on the fruit maturity processes. Interestingly, the highest correlations in mature fruits were found between phenols and pigments, particularly chlorophylls and lycopene. The results reported by other authors are variable. Studies have shown a positive direct correlation between antioxidant potential and phenolic compounds content in pepper and other crops, such as grapes, eggplants, olives, and citrus, among others [19,26,47,69]. Araujo et al. [70] reported that antioxidant activity correlated positively with several other variables, ranging from a strong to a weak correlation for chlorophyll a, chlorophyll b, titratable acidity, carotene, total phenols, flavonoids, anthocyanins, and ascorbic acid. Materska and Perucka [38] highlighted the influence of phenolic profile and its relation to the number and positions of hydroxyl groups in aromatic rings, esterification or the free form of the analyzed compounds, and methoxy substituents in the ortho position to OH. However, Chavez-Mendoza et al. [1] reported an inverse correlation between antioxidant capacity and lycopene.

Finally, as the four groups formed by the PCA analysis were composed of landraces of different origins and morphological characteristics, this suggested that a wide diversity exists for the traits studied in the groups. Thus, in our collection, we identified four landraces of interest in immature fruits: P-44, which is interesting for its high content in carotenoids, chlorophylls, phenols, and ascorbic acid; P-46, with good antioxidant capacity and high lycopene content; P-36 and P-43 with high phenols concentration and antioxidant capacity. However, P-48 is of much interest for its high phenols and lycopene content in red fruits. P-44 and P-41 also presented good levels of phenols, chlorophylls, and/or lycopene upon maturity. Other landraces of interest in ripe fruits were: P-39, as highlighted by its phenols, carotenoid, and DPPH values; P-35 and P-50 for their high carotenoid and lycopene contents, and excellent DPPH capacity. The PCA results generally showed the usefulness of the multivariate analysis for classification in studies with a great number of landraces and is a powerful tool in breeding programs for pepper and other crops used to describe and/or select cultivars with high added value [47,70,71,72,73].

## 4. Materials and Methods

### 4.1. Plant Material

**Figure 6:**
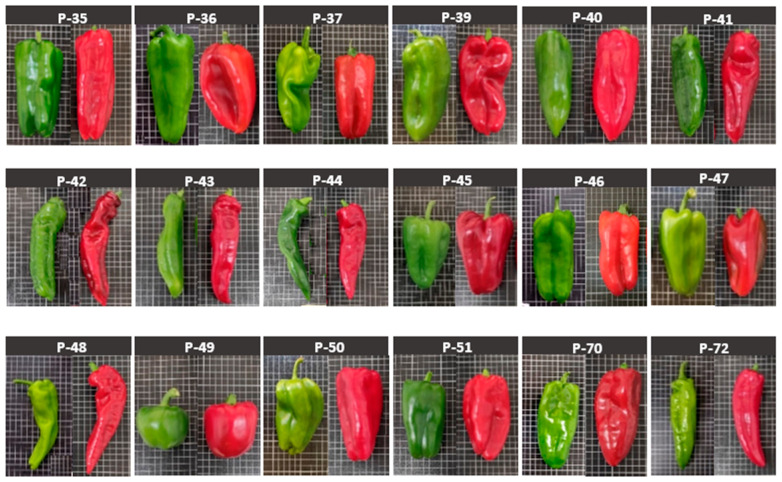
Pepper fruits in different maturity stages (red and green) obtained from the cultivated landraces. The size of the grid cells in the fruit pictures is 1 cm × 1 cm.

The plant material for this study consisted of 18 pepper landraces (*C. annuum*) that represent the pepper germplasm collection of Valencia (Spain). Landraces were provided by the COMAV and IVIA. Table 4 provides the numerical code, passport identification, fruit shape description, and origin of each landrace. Figure 6 complements this table. Seeds were sown on 7 March 2019 in 104-hole seed trays filled with enriched substrate for germination.

### 4.2. Greenhouse Experiment

The experiment was conducted from May to September 2019 in an unheated plastic multi-span greenhouse in the experimental field belonging to the IVIA (Valencia, Spain; latitude: 39.58951793357715, longitude: −0.3955507278442383). The soil composition within 20 cm depth was 68% sand, 11% clay, and 21% silt (sandy-clay loam) containing 0.61% organic matter, 0.051% total N, less than 8 mg kg^–1^ of P, 301 mg kg^–1^ of K, and 2.87 meq·100 g^–1^ of assimilable Mg. Soil electrical conductivity was 0.290 dS m^–1^ and pH was 8.1.

Plants were transplanted on 9 May 2019 and grown under greenhouse conditions in single rows (110 cm apart) with a 50 cm spacing between each plant. Each landrace consisted of six plants. Irrigation satisfied 100% of the crop evapotranspiration (ETc), as described in Penella et al. [74], performed with a drip system. Nutrients were applied by the irrigation system at a rate (kg ha^−1^) of 200 N, 50 P_2_O_5_, 250 K_2_O, 110 CaO, and 35 MgO, as recommended by Maroto [75]. The average range of minimum and maximum temperatures during the experiment was 12–24 °C for May, 15–28 °C for June, 19–32 °C for July, 19–32 °C for August, and 18–29 °C for September.

### 4.3. Nutraceutical Compounds and Antioxidant Capacity

#### 4.3.1. Sample Preparation

From each landrace, eight randomized fruits were harvested from the end of July to mid-September. Fruit samples were taken from four independent plants, four replicates for each landrace, in each maturity stage: green and red. Fruits were washed and prepared; a 3 cm-wide longitudinal section was transversally cut at the fruit midpoint and homogenized (Kinematica Polytron PT 3100, Lucerne, Switzerland) at 15,000 g for approximately 2 min. Final extracts were divided into aliquots of 0.3 and 1 g, frozen in liquid N_2_, and stored at −80 °C until further determinations.

#### 4.3.2. Total Phenolic Analysis

Phenolic content was analyzed according to Dewanto et al. [76] with modifications. Briefly, a 1 g aliquot of sample extract was homogenized in 4.0 mL of 80% (*v/v*) methanol, vortexed, incubated in an ultrasonic bath (Ultrasonic cleaner, Fungilab, Barcelona, Spain) at medium intensity for 30 min, and then revortexed. Samples were centrifuged at 10,000× *g* for 15 min at 4 °C and the supernatant was used for the analysis. Total phenolic content was determined by the Folin-Ciocalteau colorimetric method. A 10 µL aliquot of the supernatant was mixed with 115 µL of distilled water, 125 µL of Folin-Ciocalteau reagent, and 1.25 mL of NaHCO_3_ (7%) and then incubated in a dark cupboard for 90 min. Solution absorption was measured at 760 nm in a spectrophotometer (Uvikon XS, Bio-Tek, Winooski, VT, USA). The blank solution without extract was used for calibration. Each measurement was compared to a standard curve of gallic acid (GA) and total phenols were expressed as mg of GA equivalent g^−1^ FW.

#### 4.3.3. Ascorbic Acid Concentration

Ascorbic acid content was spectrophotometrically determined as described by Kampfenkel et al. [77]. Briefly, 0.3 g of each sample was homogenized and adjusted to a 2 mL volume with 6% (*w/v*) trichloroacetic acid (TCA). Samples were centrifuged at 10,000× *g* for 3 min and the supernatant was used for the analysis. Then, 0.05 mL of the homogenate was mixed with 0.05 mL of 10 mM DTT and 0.1 mL of 0.2 M phosphate buffer (pH 7.4). Samples were incubated for 15 min at 42 °C. Afterwards, 0.05 mL of 0.5% (*w/v*) NEM (N-ethylamide) was added and incubated for 1 min at room temperature. Next, 0.25 mL of 10% (*w/v*) TCA, 0.2 mL of H_3_PO_4_ 4% (*w/v*), 0.2 mL of 2-2’-dipyridyl, and 0.1 mL of 3% (*w/v*) FeCl_3_ were added to the previous solution. They were all incubated together in a water bath for 40 min at 42 °C. Solution absorption was measured at 525 nm in a spectrophotometer (Uvikon XS, Bio-Tek, Winooski, VT, USA). Ascorbic acid was expressed as mg g^−1^ FW.

#### 4.3.4. Antioxidant Capacity Measurements

Antioxidant capacity was measured following the method reported by Brand-Williams et al. [78] with modifications. Sample extract (1 g) was homogenized in 4.0 mL of 80% methanol (*v/v*), incubated in an ultrasonic bath (Ultrasonic cleaner, Fungilab, Barcelona, Spain) at medium intensity for 30 min, and then vortexed. Samples were centrifuged at 10,000× *g* for 15 min at 4 °C and 10 μL of phenolic extract was added to 990 μL of a solution containing 3.12 × 10^−5^ M of 2,2-diphenyl-1-picrylhydrazyl (DPPH) in 80% methanol. The absorbance at 515 nm was measured against a blank solution (80% methanol without extract) after a 30-min reaction time at room temperature in the dark (optimized for the highest antioxidant concentrations in the extract) using a spectrophotometer (Uvikon XS, Bio-Tek, Winooski, VT, USA). The results were expressed as the percentage reduction of the initial DPPH absorption in the extracts and expressed as mg Trolox equivalents (TE) g^−1^ FW using a standard curve of Trolox.

#### 4.3.5. Carotenoids and Chlorophyll Concentration

Carotenoids (Car) and total chlorophyll (a and b) concentration were determined spectrophotometrically as described by Porra et al. [79]. Next, 1.5 mL of 80% acetone (*v/v*) was added to sample extracts (0.3 g) and centrifuged at 7000× *g* for 10 min. The supernatant was used for the analysis. Solution absorption was measured at 663, 648, and 470 nm in a spectrophotometer (Uvikon XS, Bio-Tek, Winooski, VT, USA). Then, 80% acetone (*v/v*) was used as the blank solution (without extract). The chlorophyll (a and b) and carotenoids content of the extracts were calculated using the following equations:

Chl a = 12.25 × Abs663 − 2.55 × Abs648 (µg mL^−1^) (1)

Chl b = 20.31 × Abs648 − 4.91 × Abs663 (µg mL^−1^) (2)

Car = [(1000 × Abs470 − 1.82 Chl a) − (85.02 × Chl b)]/198 (µg mL^−1^) (3)

Chlorophylls and carotenoids were expressed as µg g^−1^ FW.

#### 4.3.6. Lycopene Concentration

Lycopene was extracted from pepper fruit using a hexane:ethanol:acetone (2:1:1; *v:v:v*) mixture following the method of Adejo et al. [80] with modifications. Sample extract (10 mg) was dissolved in 1 mL of distilled water and vortexed in a water bath at 30 °C for 1 h. Then, 8.0 mL of the hexane, ethanol, and acetone mix was added, capped, and revortexed, followed by incubation in a dark cupboard for 60 min. Subsequently, 1 mL of distilled water was added to each sample, vortexed once more, and left until it separates into phases. Care was taken to ensure that the formed bubbles had fully disappeared. The cuvette was rinsed with the upper layer of one of the blank samples before using more fresh blank samples (distilled H_2_O without extract) to zero the spectrophotometer at 503 nm. Three milliliters of the upper layers of the lycopene samples was taken and their absorbance at 503 nm wavelength was read by a spectrophotometer (Uvikon XS, Bio-Tek, Winooski, VT, USA). The lycopene content of extracts was expressed as mg g^−1^ FW.

### 4.4. Statistical Analysis

The results for the nutraceutical compounds and antioxidant capacity parameters were subjected to a one-way analysis of variance (ANOVA) using Statgraphics Centurion XVII (Statistical Graphics Corporation 2014) with landrace taken as the factor of the analyses. Each ripening state (green and red fruits) was separately analyzed. The results were expressed as mean ± standard deviation. Means were accepted as being significantly different at a 95% confidence interval (*p* ≤ 0.05). The mean, maximum and minimum values, coefficient of variation, and F-ratio of the nutraceutical traits in green and red fruits were calculated. An analysis of the correlation between the different traits in each ripening state was calculated as the linear correlations between the individual samples of each accession (*n* = 72) and the correlation coefficient (r) was obtained.

A principal component analysis (PCA) using Statgraphics Centurion XVII (Statistical Graphics Corporation 2014) was carried out for the standardized values using pairwise Euclidean distances among landraces means to assess the relations between genotypes. The correlation coefficients for each fruit trait for the first three principal components (PCs), the extracted eigenvalues, and relative and cumulative proportions of total variance explained by these components, were calculated. Two-dimensional (2D) scatter plots (first vs. second in the green fruits, and first vs. second and first vs. third in red fruits) were prepared based on a distance matrix for the PCs to visualize the relationship explaining the traits. From the PCs scatter plots, four groups of accessions were established for each ripening state with a different profile for the studied traits. Groups A (positive for both components, +/+), B (+/−), C (−/−), and D (−/+) were formed. The signification of differences among groups of landraces was evaluated by a one-way ANOVA.

## 5. Conclusions

From the analysis of the nutraceutical compounds of 18 pepper landraces, we conclude that:

(1) Landrace type and harvest pepper period can be chosen to achieve the desired optimal fruit quality. Mature fruits are related to high vitamin and carotenoids contents, for which landrace P-39 is remarkable, while green ones are associated with high polyphenol contents, traits that highlight the importance of accession P-44;

(2) Nutritional characterization of pepper landraces can contribute to promote their use and increase their added value. This work could be of practical use as a start point in breeding programs for growing antioxidant-rich varieties, especially with good levels of vitamin C and total phenolics, and for enhancing the conservation of traditional varieties that are in danger of genetic erosion.

## Figures and Tables

**Table 1:** Variation parameters for quality traits in 18 pepper landraces cultivated in Spain.

Phytochemicals Concentration	Unit/Scale	Mean	Range	CV (%)	F-Ratio
Green Fruit					
Phenols	mg g^−1^ FW	4.17 ± 1.43	1.83–7.24 ***	34.32	2.59
Ascorbic acid	mg g^−1^ FW	1.44 ± 0.40	0.60–2.47 ***	27.93	3.65
Lycopene	mg g^−1^ FW	0.62 ± 1.4	0–8.25 **	225.06	2.46
Carotenoid concentration	µg g^−1^ FW	6.35 ± 2.15	2.64–13.11 *	33.87	2.12
Chlorophyll a + b	µg g^−1^ FW	35.07 ± 10.97	14.85–62.82 **	31.29	2.85
Antioxidant capacity	mg TE g^−1^ FW	36.57 ± 17.88	6.12–77.84 ***	48.91	8.31
Red Fruit					
Phenols	mg g^−1^ FW	9.20 ± 1.97	5.66–15.87 ***	21.45	3.20
Ascorbic acid	mg g^−1^ FW	2.24 ± 0.66	1.05–3.94 ***	29.86	3.73
Lycopene	mg g^−1^ FW	16.98 ± 10.11	0.42–49.28 ***	59.51	15.50
Carotenoid concentration	µg g^−1^ FW	30.58 ± 14.05	12.17–103.88 ***	45.95	3.38
Chlorophyll a + b	µg g^−1^ FW	1.18 ± 1.10	0–5.31	93.18	1.46
Antioxidant capacity	mg TE g^−1^ FW	83.92 ± 12.4	49.68–96.31 ***	14.77	19.42

Values represent the mean, range, significance (***, **, * indicate significance at *p* < 0.001, *p* < 0.01, *p* < 0.05, respectively), coefficient of variation (CV %), and F-ratio for the quality traits studied.

**Table 2:** Correlation coefficients for each morphological trait for the three first principal components, eigenvalue, and the relative and cumulative proportions of total variance explained by these components, in the collection of the 18 pepper landraces.

	Component 1	Component 2	Component 3
**Green Fruits**			
Phenols	0.385	0.458	
Ascorbic acid	0.541	0.050	
Lycopene	−0.247	0.357	
Carotenoid	0.575	−0.027	
Chlorophyll a + b	0.384	−0.499	
Antioxidant capacity	0.142	0.640	
Eigenvalue	2.60	1.73	
Variance explained (%)	43.32	28.82	
Cumulative variance explained (%)	43.32	72.14	
**Red Fruits**			
Phenols	0.602	0.136	−0.155
Ascorbic acid	0.239	−0.658	−0.220
Lycopene	0.483	0.268	0.379
Carotenoid	0.201	−0.316	0.807
Chlorophyll a + b	0.464	0.350	−0.269
Antioxidant capacity	0.303	−0.504	−0.245
Eigenvalue	2.02	1.22	1.03
Variance explained (%)	33.59	20.31	17.09
Cumulative variance explained (%)	33.59	53.90	70.99

**Table 3:** Mean values for the fruit traits in the four groups of accessions established by a multivariate PCA in fruits of the collection of 18 pepper landraces. ***, **, *, and ns indicate significance at *p* < 0.001, *p* < 0.01, *p* < 0.05, and non-significant values, respectively. Different letters in each trait indicate significant differences between groups. at *p* < 0.05 using the LSD test.

**Green Fruits**																	
**First/Second PC**																	
	Group																
	A				B				C				D				
Phenols	5.51	±	1.43	a	3.84	±	1.35	b	3.46	±	0.98	b	4.21	±	1.23	b	***
Ascorbic acid	1.66	±	0.31	a	1.70	±	0.45	a	1.20	±	0.33	b	1.34	±	0.29	b	***
Lycopene	0.50	±	0.65	b	0.20	±	0.41	b	0.14	±	0.32	b	1.86	±	2.49	a	***
Carotenoid	7.80	±	2.49	a	7.11	±	1.80	a	5.36	±	1.87	b	5.63	±	1.45	b	***
Chlorophyll a + b	35.92	±	11.61	ab	42.56	±	10.05	a	34.36	±	9.89	bc	28.20	±	8.56	c	**
DPPH	51.89	±	10.18	a	27.07	±	19.11	a	26.55	±	13.03	b	46.12	±	12.97	b	***
**Red Fruits**																	
**First/Second PC**																	
	Group																
	A				B				C				D				
Phenols	10.04	±	2.13	a	10.00	±	1.60	a	7.77	±	1.08	b	8.86	±	1.96	ab	***
Ascorbic acid	1.96	±	0.58	b	2.68	±	0.62	a	2.27	±	0.64	ab	2.09	±	0.69	b	**
Lycopene	24.96	±	10.56	a	14.60	±	10.12	b	10.01	±	4.76	b	15.82	±	3.23	b	***
Carotenoid	32.01	±	12.24	ab	35.85	±	12.85	a	25.23	±	6.01	b	23.93	±	7.54	b	**
Chlorophyll a + b	1.60	±	1.04	a	1.51	±	1.50	a	0.53	±	0.64	b	1.10	±	0.69	ab	**
DPPH	83.18	±	12.99	b	93.57	±	3.86	a	86.61	±	6.72	b	65.90	±	8.50	c	***
**Red Fruits**																	
**First/Third PC**																	
	Group																
	A				B				C				D				
Phenols	9.52	±	1.76	b	10.78	±	1.94	a	8.17	±	1.03	c	8.20	±	2.20	c	***
Ascorbic acid	2.07	±	0.70		2.54	±	0.59		2.26	±	0.65		2.11	±	0.67		ns
Lycopene	22.97	±	7.17	a	17.59	±	15.64	ab	10.65	±	5.55	c	14.76	±	2.80	bc	***
Carotenoid	38.03	±	12.48	a	26.95	±	9.36	b	23.58	±	6.50	b	26.67	±	6.40	b	***
Chlorophyll a + b	1.33	±	0.86	a	1.88	±	1.58	ab	0.80	±	0.73	b	0.66	±	0.69	b	**
DPPH	84.49	±	13.45	ab	91.28	±	6.71	a	83.49	±	11.97	b	70.54	±	8.00	c	***

**Table 4:** Abbreviation, germplasm collection code, fruit shape description, and origin of the 18 pepper varieties included in the study. Plant material was provided by: (1) the Valencian Institute for the Conservation and Improvement of Agrobiodiversity (COMAV, Spain) and (2) the Valencian Institute for Agricultural Research (IVIA, Spain).

Code	Germplasm Code	Fruit Description	Origin
P-35	BGV005087(1)	Rectangular shape, blocky and with four shoulders, and locule marked	Fanzara, Castellón, Spain
P-36	BGV005035(1)	Irregular, rectangular-conical shape but inconsistent pattern, slightly marked shoulders	Chelva, Valencia, Spain
P-37	BGV005097(1)	Triangular shape and truncated apex	Castillo de Villamalefa, Castellón, Spain
P-39	BGV005115(1)	Triangular shape and truncated apex	Alicante, Spain
P-40	BGV005125(1)	Rounded-elongated triangular shape and truncated apex	Elda, Alicante, Spain
P-41	BGV014141(1)	Elongated (horn type), very slightly marked shoulders	Vinaròs, Castellón, Spain
P-42	BGV014145(1)	Elongated (horn type), very slightly marked shoulders	Almenara, Castellón, Spain
P-43	BGV014146(1)	Elongated (horn type), very slightly marked shoulders	Castellón de la Plana, Castellón, Spain
P-44	BGV016188(1)	Elongated (horn type), very slightly marked shoulders	Guardamar del Segura, Alicante, Spain
P-45	BGV005064(1)	Triangular shape and truncated apex	Ademuz, Valencia, Spain
P-46	BGV005085(1)	Rectangular shape, blocky and with four shoulders, and locule marked	Onda, Castellón, Spain
P-47	BGV005040(1)	Rounded-elongated triangular shape and truncated apex	Siete Aguas, Valencia, Spain
P-48	BGV005034(1)	Elongated (horn type), very slightly marked shoulders	Chelva, Valencia, Spain
P-49	BGV005046(1)	Ball-like shape with very slightly marked shoulders	Benissa, Alicante, Spain
P-50	BGV005116(1)	Triangular shape and truncated apex	Rojales, Alicante, Spain
P-51	BGV014553(1)	Rectangular shape, blocky and with four shoulders, and locule marked	Tales, Castellón, Spain
P-70	IVIA 70(2)	Rounded-elongated triangular shape and apex truncated	Moncada, Valencia, Spain
P-72	IVIA 72(2)	Elongated (horn type), very slightly marked shoulders	Canal de Navarrés, Valencia, Spain

## Data Availability

Not applicable, as the study did not involve humans or animals.

## Sample Availability

Samples of the compounds are available from the authors.
